# Pediatric Benign Fibro-Osseous Lesions of the Nose and Paranasal Sinuses: A Tertiary Hospital Experience

**DOI:** 10.1155/2022/1608015

**Published:** 2022-08-25

**Authors:** Dalia Al Arfaj, Haifa Lafi Alenzi, Ali Almomen, Musab Bakri

**Affiliations:** ^1^Department of Otorhinolaryngology Head and Neck Surgery, King Fahad Specialist Hospital, Dammam, Saudi Arabia; ^2^Department of ENT, North Medical Tower, Arar, Saudi Arabia; ^3^Department of Radiology, King Fahad Specialist Hospital, Dammam, Saudi Arabia

## Abstract

**Background:**

Pediatric benign fibro-osseous lesions of the nose and paranasal sinuses are considered rare neoplastic entities. These fibro-osseous lesions are difficult to accomplish owing to the multifaceted anatomy of the skull base in addition to the closeness to neurovascular configurations.

**Objective:**

The study aimed to study different clinical presentations, radiological manifestations, surgical management, and consequences of different benign fibro-osseous lesions in the pediatric age groups. *Methods and Settings*. This is a case series study of a single-center experience.

**Results:**

Four different cases were presented and discussed (osteoma, fibrous dysplasia, and ossifying fibroma.

**Conclusion:**

We reported four different cases of osteoma, ossifying fibroma, and fibrous dysplasia. All of these cases were managed endoscopically with no postoperative complications. Endoscopic management is considered highly effective with reduced morbidity. Preoperative radiographic studies are highly essential for diagnosis and operative planning.

## 1. Introduction

“A group of diseases affecting the paranasal sinuses and anterior skull base are collectively referred to as “fibro-osseous lesions,” and they are distinguished by the substitution of natural bone with varying numbers of fibroblasts. A diverse category of illnesses known as benign fibro-osseous disorders of the maxillofacial skeleton comprises developmental, reactive (dysplastic), and neoplastic lesions. The utmost prevalent benign fibro-osseous disorders comprise fibrous dysplasia, osseous dysplasia, aneurysmal bone cysts (ABCs), calcifying fibroma, and juvenile ossifying fibromas, despite the fact that their categorization has indeed been revised numerous times over the years (JOFs) [1–3].

These fibro-osseous lesions are difficult to accomplish owing to the multifaceted anatomy of the skull base in addition to the closeness to neurovascular configurations. It is also challenging for physicians to differentiate between lesions due to the significant overlay of histologic and radiographic types in these conditions. These tumors could only really be recognized by their growth decoration, which is visible on the face and head CT scans or conventional radiography [4].

Osteoma remains the greatest shared category, shadowed by fibrous dysplasia and then petrifying fibroma [5]. Bone dysplasia as well as neoplasm tumors are the two groups of fibro-osseous disorders that may be distinguished. Fibrous dysplasia is by far the most widespread bone abnormality in children. Benign neoplastic abnormalities called JOFs are marked by rapid proliferation. Benign bone-producing fibrous neoplasia of such bones known as an ossifying fibroma might develop in any cheekbone, but it is much more prevalent in the mandible than in the other bones of the face. Whenever sufferers remain younger, clinicians refer to the tumor as a juvenile ossifying fibroma. The term “ossifying fibroma” refers to a group of tumors that could vary in their overall clinical manifestations, location of preference, gender, age structure, and micromorphology. Due to this, osseous fibroma could be distinguished as fibrous dysplasia [6].

Fibrous dysplasia could be monostotic or single bone, or else polyostotic meaning multiple bones. It is characterized by the normal bone marrow, and cortical bones are substituted with immature fibro-osseous tissue intermixed with woven bone. Craniofacial contribution of fibrous dysplasia is frequent, with 10% to 27% of monostotic patients and up to 50% of polyostotic patients having an association with one or more craniofacial bones. Fibrous dysplasia is initiated mainly by a mutation in GNAS 1*α* [7, 8]. The lesions' radiographic unique features depend on the condition's phase. Initial tumors are frequently luminescent, becoming increasingly opaque as they calcify. Craniofacial tumors frequently have a radiolucent/radiopaque mixture, giving them the impression of a “ground glass.” This lesioned bone mixes imperceptibly with the surrounding normal-appearing bone because the borders are imprecise. It is possible for the paranasal sinuses to disappear, as well as the orbit to be frequently moved. To make an accurate diagnosis and develop a clear therapeutic approach, relevant medical, radiologic, and microscopic aspects must be carefully correlated. For a proper diagnosis, it is crucial to consider the demography, medical examination, operational presentation, macroscopic attractiveness, and, more critically, the histological and radiography features [2].

Ossifying fibroma lesions can be distinguished from conventional fibrous dysplasia by 2 primary features. Well, first of all, it has been established that lesions do not include the GNAS 1 mutation. Furthermore, postcranial lesions are typically absent in individuals with this fibro-osseous lesion [9, 10].

Based on clinical appearances, radiographic characteristics, and related morbidity, these lesions are treated. Owing to such tumors' intricate anatomical positions inside the orbits and skull base, as well as the growing propensity that encircles the nearby neurovascular pathways, surgical excision and histopathological examination of such tumors are frequently difficult [3].

### 1.1. Aim and Objective

The study aimed to explore different clinical presentations, radiological manifestations, surgical management, and outcomes of different benign fibro-osseous lesions in the pediatric age groups.

## 2. Methodology

### 2.1. Study Design

This is a case series study of a single-center experience.

### 2.2. Study Population

The study included all the pediatric patients diagnosed with benign fibro-osseous lesions of the nose and paranasal sinuses in a tertiary referral hospital, King Fahad Specialist Hospital, Dammam, KSA. The study did not follow up nor perform survivorship analysis. However, for full disclosure, the clinicopathological records and management plans applied to the included subjects are reported.

### 2.3. Ethical Considerations

The hospital's ethics board permitted us to access all relevant medical files, office papers, pictures, and surgical and pathological reports. All operations were carried out in accordance with the ethics committee's recommendations regarding patient confidentiality. Age, sex, symptoms, tumor location and size, as well as a management plan, were all retrieved.

## 3. Results

The study included four patients that were diagnosed with four different benign fibro-osseous lesions. Among four patients, three (75%) were males. The age group of cases was from 4–15. The mean age (±SD) was 9.2 (±5.8). The pathologies diagnosed were osteoma (25%), fibrous dysplasia (50%), and ossifying fibroma (25%).

**Figure 1:**
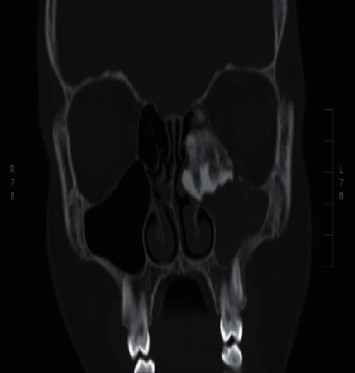
The CT scan of the paranasal sinuses shows a left ethmoid mass.

**Figure 2:**
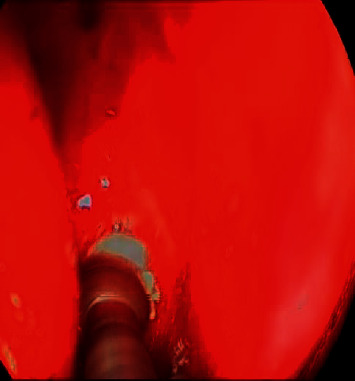
The intraoperative endoscopic vision of the leftward sphenoid bulk preoperative and postoperative.

**Figure 3:**
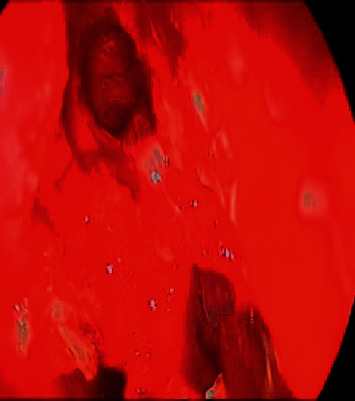
The intraoperative endoscopic view of the left sphenoid mass preoperative and postoperative.

An osteoma was diagnosed in a 14-year-old male (patient ^#^1) who presented to the clinic complaining of a chronic left-sided headache and left-sided nasal obstruction. He denied any history of epistaxis, allergic rhinitis, or sinusitis. Nasal endoscopy was performed and showed a mass lateral to the middle turbinate in the left nasal cavity with no other findings. A left ethmoid tissue mass having inferior expansion further into the superior portion of such a left maxillary antrum was visible on a CT scan of such a maxillary sinus. It really has calcification densities just at the perimeter as well as heterogeneous densities that resemble ground glass. The lateral aspect of the mass results in the renovation of the leftward medial orbital partition (Figure 1). The patient was managed by image-guided endoscopic removal of the mass on or after the leftward ethmoid with orbital and base of the skull preservation (Figures 2 and 3). He was followed up in our clinic and was free of symptoms for two years.

**Figure 4:**
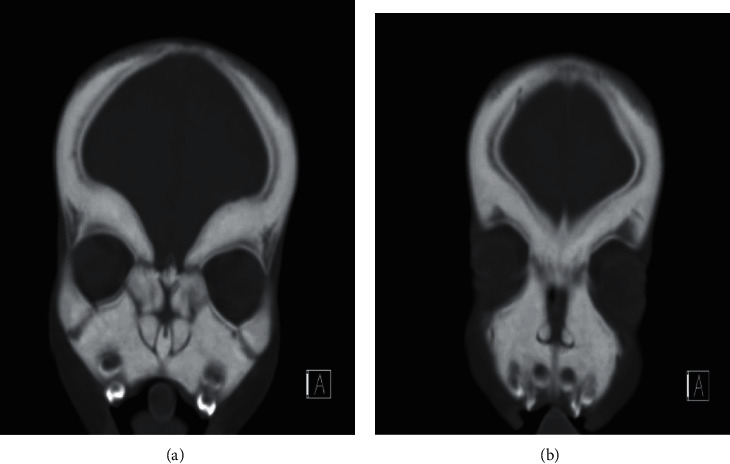
The CT scan of the brain shows extensive thickening of the facial bone.

**Figure 5:**
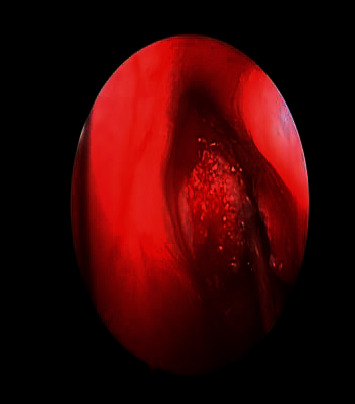
The intraoperative endoscopic image shows a bony mass filling the choana.

**Figure 6:**
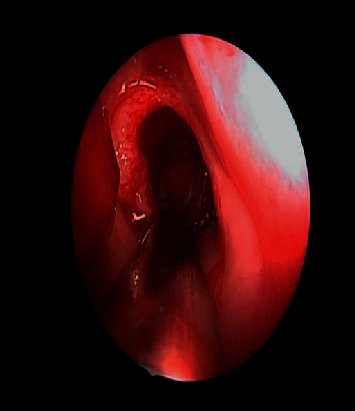
Post debulking and drilling of the choana.

Fibrous dysplasia was diagnosed in two cases; the first one is a 4-year-old male known case of craniodiaphyseal dysplasia syndrome, osteopetrosis, and developmental delay who presented to the clinic complaining of snoring, OSA, and mouth breathing. Anterior rhinoscopy showed an obstructed nasal passage by boney masses bilaterally. The CT scan of the brain showed extensive thickening and increased density of the visualized bones with no sparring, which resulted in obliteration and narrowing of the nasal cavity and paranasal sinuses that are suggestive of fibrous dysplasia (Figures 4). The patient underwent endoscopic drilling of all boney masses obstructing the nasal passage bilaterally till reaching and creating patent choana (Figures 5 and 6). The patient is still following up in our clinic maintaining a patent nasal passage for 3 years postoperative.

**Figure 7:**
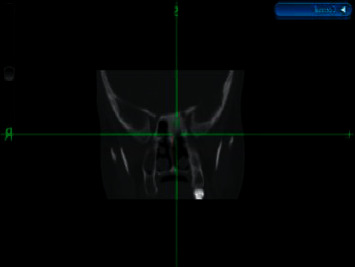
Imaged-guided nasal endoscopy shows a left sphenoid sinus lesion.

The other fibrous dysplasia case was diagnosed in a 5-year-old boy complaining of left retro-orbital pain and headache. No other symptoms were appreciated. The nasal endoscopic exam was normal. A CT scan of the nose and paranasal sinuses demonstrated expanded left sphenoid bone with ground-glass attenuation and areas of mixed sclerosis and lucencies consistent with fibrous dysplasia. The patient underwent image-directed endoscopic debulking of the found mass (Figure 7). No postoperative complications were noticed. The final pathology report was suggestive of fibrous dysplasia. The patient remained symptoms free, with no signs of recurrence for 2 years of follow-up.

A juvenile ossifying fibroma was diagnosed in a 15-year-old girl, medically free, presented to our clinic complaining of a right-hand side worsening eye condition, right-sided intranasal blockage, and proptosis over 3 month duration. The patient did not experience any discomfort, headache, visual abnormalities, or diplopia. She did not have any symptoms of allergic rhinitis or sinusitis.

**Figure 8:**
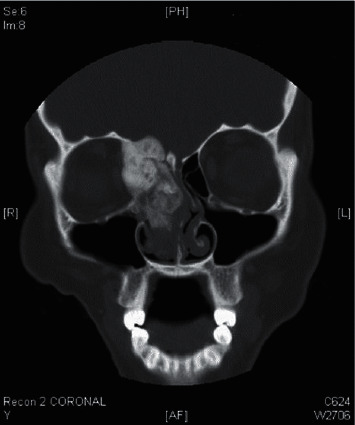
The right ethmoid sinus and nasal space growth encompassing penetration of the orbital as well as cerebral interplanetary are perceived preceding the paranasal coronal CT scan image.

**Figure 9:**
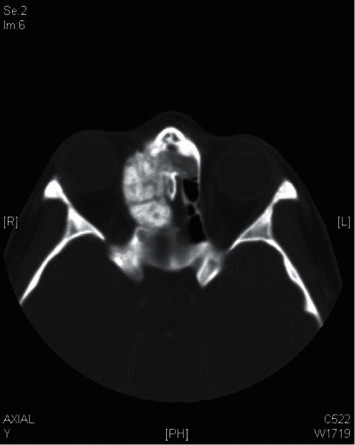
The paranasal axial CT scan demonstrates the right ethmoid sinus besides nasal hollow bulk by orbital then intracranial engrossment.

**Figure 10:**
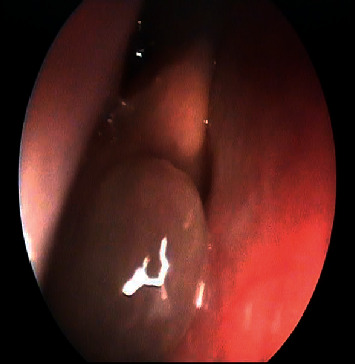
The intraoperative endoscopic spitting images illustrate various nasal polyps.

Examination revealed right-sided eye proptosis. Nasal endoscopy showed right-sided multiple nasal polyps with a deviated nasal septum to the left (Figure 8). The CT scan of the nose and paranasal sinuses demonstrates a large, calcified mass centered at the right orbital plate. The mass is expansile with heterogeneous density and ground glass appearance, associated with remodeling of the surrounding bony margins, the mass has spread to the right ethmoid cavity with obliteration of the right ostiomeatal complex, and proptosis of the right orbit is noted (Figure 9). This patient had a number of procedures, comprising debulking of the tumor, laterally rhinotomy, and frontal craniotomy including restructuring, which were all accomplished in collaboration with such a neurosurgeon staff (Figure 10). Postoperative paranasal coronal CT scan with aerated sinuses is shown in 11.

The above patient underwent a variety of procedures with the support of a neurosurgery crew, involving debulking of the tumor, unilateral rhinotomies, and frontal craniotomies involving reconstruction.

## 4. Discussion

The much more frequent benign tumor of such paranasal sinuses was osteoma. Up to a particular size, patients do not exhibit any symptoms [5]. Whenever symptoms appear, they complain of headaches, as our case finding agrees with that of Chahed et al. [11]. Other symptoms include cosmetic deformity, proptosis, epiphora, and visual complaints. They could rarely develop extensively as well as spread damagingly outside of the Sino nasal canal, leading to serious and potentially existence consequences. They are commonly observed in the third to fourth decades of life and thus are considered rare in the pediatric population. The most frequent presentations, according to Sinha et al., occurred in the second and the third decades [12]. The age disparity has been related to ethnocultural disparities [13]. According to research conducted by Sooknundan, Deka, and Kacker, osteomas are much more common in individuals who are likely to be younger among Indians than among Caucasians [14].

Although the cause of osteomas is unclear, suggestions including embryology, trauma, and infection have been put forth. The traumatic theory suggests that there should be a history of trauma to the face, but no causal link can be demonstrated. The basis of the infectious idea is that persistent sinusitis causes osteoma production, although this assumption is dubious. The reality that several osteomas form from the frontoethmoid seam zone, wherein membranes and cartilage tissues adjoin shortly after birth, lends support to the evolutionary idea. However, several authors have demonstrated that some do not emerge from some of these embryo connections [12].

Histopathologically, these are made up of matured, metaplastic osseous tissue that may be categorized into three categories: mixed, spongiotic, and ivory. This tissue always maintains a clear boundary with the adjacent normal structures. The much more frequently affected location is the prefrontal sinus, which is followed by the ethmoid, maxillary, and sphenoid sinuses [15]. Computed tomography (CT) is the imaging modality of choice. It commonly manifests as a well-circumscribed hyperdense and homogenous lesion in the frontoethmoidal region. The information provided by plain X-rays is typically insufficient, and the bone demarcation provided by MRI is erroneous [12].

The surgical treatment of simple sinus osteomas is debatable since it has significant possible dangers. These tumors could be effectively treated during surgeries using endoscopic, conventional, or combination procedures. The site as well as the size of the tumor affects the operative excision technique choice [16]. Endoscopic procedures could be used to securely and completely remove small and medium-sized osteomas of the paranasal sinuses. It enables their complete removal and has excellent aesthetic results. The key benefits of this approach are its minimum soft tissue deconstruction, lack of disturbance of the bones of the face, and lack of need for face incisions. A combination of exterior and internal endoscopic techniques can be used to treat giant frontal sinus osteomas [17]. According to Giotakis et al., the invasive strategic approach was used in 1 out of 5 individuals; 2 of these patients had a refined Lynch incision, and 3 used an osteoplastic flap strategy, either through a liberal constitution puncture in 2 patients or a midforehead osteotomy in 1 patient. Only two patients underwent a pure endoscope procedure, and in both cases, the enormous ethmoid osteoma was advantageously situated. Three patients received a combination technique resection in which the ethmoidal component and the portion that filled the forehead recessed region were both treated using an endoscopy method [18]. 11 patients having prefrontal sinus osteomas underwent an endoscope route, 9 underwent an exterior technique (osteoplastic flap), and 5 underwent mixed (exterior + endoscopic) reach. All ethmoid osteoma sufferers favored an endoscopy technique. One patient with a maxillary sinus osteoma had a Caldwell–Luc surgery combined with an endoscopy technique [19]. In contrast to the 39 open operations that were carried out and the combination method that was employed in one case, Chahed et al. [11] disclosed the excision of 4 osteomas with endoscopy help. According to Deger et al., there are a number of grade systems that can help with surgery technique selections, but clinical features and operative implications must not be overlooked while monitoring individuals with a frontal sinus osteoma. The surgical operation may be started endoscopically and, if essential, and if the subject is told beforehand, it can be supplemented with just an exterior technique whether there are worries about the grade of the osteoma, and the endoscopic method is thought to be insufficient for resection [20].

A profoundly chaotic combination of immature fibrous connective tissue and pieces of immature trabecular bone characterizes fibrous dysplasia, a developing defect of the bone. It can manifest as just a solitary, isolated (monostotic) tumor, and just as many lesions affecting numerous bones, which would be more common (oligo- or polyostotic) [21].

Ninety percent of the bones involved in craniofacial FD manifest even before the age of five and include numerous bones. The hazard of sarcomatous deterioration exists despite the rarity of malignancy (less than 1%), and it is heightened by occupational exposure. Typically, histopathologic, radiological, and clinical characteristics are used to make the diagnosis of FD [22]. Medically, as documented in both instances and other initially disclosed cases, the much more frequent presentation is unusual discomfort in the head and face together with associated symptoms of sinusitis [23, 24]. Many FD instances are unintentionally found on regular dental or facial X-ray images whenever sufferers present with symptoms unconnected with this illness. Together with the characteristics of FD, sufferers could also develop mucocele complaints, whereby the bony changes of the disease process caused obstruction or dysfunction to the ostium [25, 26].

A hazy, radiolucent, or ground-glass structure caused by the improper calcification of young hypoplastic bones is the hallmark radiological sign of fibrous dysplasia. This arrangement is typically remarkably distinct from the radiological image of bone structure, calcified cartilage, or soft tissue. Radiology and computed tomography (CT) imaging of fibrous dysplasia in the neck and head reveal a vaguely understood tumor that blends with nearby bone [22]. In these situations, radiological confirmation is typically adequate, negating the need for further bone biopsy. Clinical observations of lesions that represent low chances of pathologic fractures or deformities can be made based on the site, the patient's age, the kind of FD, and their attitudes regarding operation. Early lesions may be radiolucent, but they become increasingly radiopaque and typically show a diffuse radiopacity or “ground-glass” appearance [23].

The best way to treat FD depends frequently on the unique presentation of each patient. Excision of bothersome tumors, avoidance or repair of pathogenic fracture, and rectification of undesirable aesthetic or practical abnormality are a few of the justifications for operation after confirming biopsy. A further crucial factor is the current age of manifestation. The operative method might be as conservative as curettage, bone remodeling, and contouring, or as drastic as complete excision of the affected bone followed by rapid repair. The best course of therapy is intensive surgery since it allows for the entire excision of tumors [21, 22].

We discussed two FD instances, one of which included the nasopharynx and the second of which involved the sphenoid sinus. The bilateral nasal blockage was the primary complaint of FD of the nasopharynx, whereas headaches and advanced or recurrent discomfort were the primary complaints of FD of the sphenoid sinus. Both of these patients were handled endoscopically and without any difficulties following the procedure. Total endonasal endoscopic excision of the lesion was described by Shkarubo et al., and control SCT was used to corroborate their findings. As the neurological state maintained at its pretreatment state, the straight exophthalmos slightly receded [23]. In an instance of infantile FD of the paranasal sinuses, sphenoidotomy was performed using a Lynch incision through the left ethmoid cells, according to Feldman et al. [24]. According to Xiong et al., a conventional imaging evaluation on a 28-year-old lady with nasopharyngeal cancer and fibrous dysplasia of the bone revealed the existence of a single right femur metastasis from NPC. After four cycles of chemotherapy, the patient's neck mass showed a partial response (PR), but the bone metastases remained unaffected. As there was just one bone lesion, a histopathological sample was performed to validate the diagnosis. Rather than just a tumor, there was unexpectedly an FDB [27].

Pediatricians classify juvenile calcifying fibroma as a heterogeneous, harmless fibro-osseous tumor of the craniofacial skeleton. It develops when a healthy bone is supplanted by something like a fibrous cellular stroma that contains mineralized or calcified foci [28].

The jaw and zygomatic arch are by far the most common locations for osseous fibromas. They have rarely been reported to develop in the orbits and ethmoid sinuses [29]. Since periodontal ligaments of teeth have the ability to manufacture cementum and osteoid material, it is assumed that these lesions are the result of OF, despite the fact that its genesis is unclear. Trauma and cognitive reasons are some more hypotheses [30].

People with OF exhibit more combative behavior earlier in the process, from infancy to the eighth decade of life. Here, between the ages of 20 and 40, there is a higher than average prevalence of OF, with a female preference [31]. Complaints such as nose blockage, anosmia, hyposmia, headache, or epistaxis are brought on by the aggregate impact of sinonasal tumors. Visual loss, diplopia, proptosis, and epiphora, just like in our case, are examples of ocular complaints [31]. Calcifying fibromas, albeit harmless tumors, have a significant potential to infiltrate nearby tissues, including the orbits, which can cause a variety of indications and symptoms depending on the squeezed components [32].

The radiometric findings that reveal the placement, thickness, and/or damaged and/or supporting structures of sinonasal tract ossifying fibromas are crucial to their management. According to radiographic descriptions, JOF is a large mass with sclerotic boundaries that is locally aggressive and damaging to the cortex [33]. JOF is surgically removed, but if the local resection is incomplete, it may return. There has never been a report of clear-view operation to remove an ossifying fibroma with intraorbital infiltration in the sinonasal canal [34].

Consequently, whether or not a whole resection can be accomplished will determine whether to use an open operating technique using a transfacial, transoral, or craniotomy rather than an endoscopic or a mix of open and endoscopic endonasal approach [35]. According to the available information, the juvenile ossifying fibroma (JOF), which is obviously more aggressive, should always be completely removed, but for the asymptomatic adult paranasal ossifying fibroma (COF), a wait-and-scan strategy may be chosen, as is advised for paranasal osteoma and fibrous dysplasia [36].

When performed by a skilled surgeon, endoscopic excision of sinonasal OF is a fantastic treatment option. Direct vision, magnification, the absence of outward deformity, and reduced morbidity are benefits. A skull base injury complication that results in cerebrospinal fluid leaking might be immediately treated by using an endoscope [37].

In this case study, we described a right ethmoid cavity complex with active juvenile ossifying fibroma with the involvement of the orbit and skull base. The lesion was debulked, a lateral rhinotomy was performed, and a frontal craniotomy with reconstruction was all used to treat it. To ensure there was no recurrence, the patient was followed up. The majority of instances of ossifying fibroma in the sinonasal tract, including the ethmoid sinus and the orbit, have manifested with little to no intraorbital involvement, despite the fact that there are just a few examples recorded [31, 38–40]. In one case series, the majority of patients were said to be asymptomatic or to have only minimal symptoms, such as nasal blockage [41]. The magnitude of the tumor's impact on the optic nerve caused vision loss owing to compression, according to Ta, et al., was a crucial worry [42].

## 5. Conclusion

Pediatric benign fibro-osseous lesions of the nose and paranasal sinuses are considered rare neoplastic entities. We reported 4 different cases of osteoma, ossifying fibroma, and fibrous dysplasia. All of these cases were managed endoscopically with no postoperative complications. Endoscopic management is considered highly effective with reduced morbidity. Preoperative radiographic studies are highly essential for diagnosis and operative planning.

## Figures and Tables

**Figure 11:**
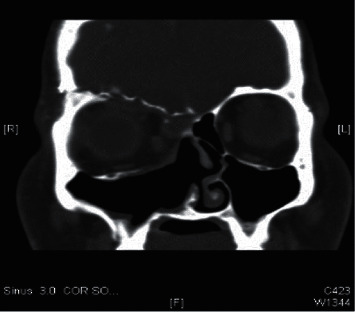
Postoperative paranasal coronal CT scan.

## Data Availability

The data used to support the findings of this study are included within the article and are available from the corresponding author upon request.
